# Extremophiles, a Nifty Tool to Face Environmental Pollution: From Exploitation of Metabolism to Genome Engineering

**DOI:** 10.3390/ijerph18105228

**Published:** 2021-05-14

**Authors:** Giovanni Gallo, Rosanna Puopolo, Miriam Carbonaro, Emanuela Maresca, Gabriella Fiorentino

**Affiliations:** 1 Department of Biology, University of Naples Federico II, Via Cinthia 21, 80126 Napoli, Italy; giovanni.gallo2@unina.it (G.G.); rosanna.puopolo@unina.it (R.P.); miriam.carbonaro@unina.it (M.C.); emanuelamaresca95@gmail.com (E.M.); 2 Consiglio Nazionale delle Ricerche CNR, Institute of Polymers, Composites and Biomaterials (IPCB), Via Campi Flegrei, 34, 80078 Pozzuoli, Italy

**Keywords:** extremophiles, environmental pollution, heavy-metal resistance, aromatic-compounds, bioremediation, biosensors, genome-engineering, CRISPR-Cas

## Abstract

Extremophiles are microorganisms that populate habitats considered inhospitable from an anthropocentric point of view and are able to tolerate harsh conditions such as high temperatures, extreme pHs, high concentrations of salts, toxic organic substances, and/or heavy metals. These microorganisms have been broadly studied in the last 30 years and represent precious sources of biomolecules and bioprocesses for many biotechnological applications; in this context, scientific efforts have been focused on the employment of extremophilic microbes and their metabolic pathways to develop biomonitoring and bioremediation strategies to face environmental pollution, as well as to improve biorefineries for the conversion of biomasses into various chemical compounds. This review gives an overview on the peculiar metabolic features of certain extremophilic microorganisms, with a main focus on thermophiles, which make them attractive for biotechnological applications in the field of environmental remediation; moreover, it sheds light on updated genetic systems (also those based on the CRISPR-Cas tool), which expand the potentialities of these microorganisms to be genetically manipulated for various biotechnological purposes.

## 1. Introduction

Natural environments on Earth display the most miscellaneous life conditions, and microorganisms are among the few entities that are able to grow in very extreme and inhospitable habitats. Hot springs, volcanic areas, polar regions, saline-alkaline, or acidic lakes and deep-see hydrothermal vents are some examples of natural environments that show temperature, salt concentration, pH, and pressure conditions very harsh for almost all forms of life [1].

Extremophiles are microorganisms that can live in these kinds of natural niches, and based on the conditions in which they thrive, they can be grouped in: acidophiles/alkaliphiles that grow at acid or alkaline pHs, halophiles that can live at high salt concentrations, piezophiles that prosper in high pressure conditions, metallophiles that are able to thrive in presence of metals/heavy metals, psychrophiles which live at low temperatures, and thermophiles/hyperthermophiles that grow at elevated temperatures [2]. Moreover, as a result of anthropogenic activities, some microbes adapted to flourish in polluted environments such as industrial wastewaters and contaminated soils characterized by the presence of toxic substances like pesticides, heavy metals, and different chemicals [3,4]. Therefore, extremophiles possess peculiar biological molecules and metabolic pathways that allow them to face multiple environmental stresses, sometimes simultaneously. For example, enzymes of thermophiles (called thermozymes), in comparison to their mesophilic counterparts, maintain their activity and their folding at higher temperatures due to a more compact hydrophobic core, and a better distribution of hydrogen bonds and salt bridges at the protein surface [5]. At the same time thermozymes are also stable in the presence of organic solvents, denaturing agents, and high salinity, and more resistant to proteolysis, mirroring the niches where they are found. So far, extremophiles have increasingly received attention for their biotechnological significance and for industrial purposes; the study of these peculiar microorganisms, their metabolisms and catalysts makes possible to develop bioremediation technologies and bio-based energy processes.

In the last years, as a consequence of environmental pollution, global warming, and depletion of non-renewable sources and with the push of the 2030 Agenda for Sustainable Development drawn up by the United Nations [6], many research efforts have been focused either on the optimization of green and sustainable industrial processes (biorefineries), and on the setup of biotechnological methods to monitor and remove pollutants from the environment (biomonitoring and bioremediation, respectively) [7,8]. In this context, investigation on the biology, ecology, and physiology of microorganisms is a necessary prerequisite to set up white and green biotechnologies [9] in the field of industrial processes, energy generation [10], prevention of environmental pollution by detection and/or removal of contaminants [11], and production of biopolymers from renewable resources [12,13]. For example, thermophilic microorganisms can find applications to reduce pollution in industrial wastewaters that are often characterized by higher temperatures and highly dissolved heavy metals [14,15]. Moreover, thermophiles are more advantageous than mesophiles in biorefineries which require high-temperature steps [16].

This review analyses the metabolic strategies adopted by extremophilic microorganisms, with major emphasis on thermophilic ones, to face three classes of compounds with high impact on environmental pollution: heavy metals, organic compounds, and lignocellulosic biomasses, as well as their exploitation for application in bioremediation, biosensing, and biorefinery. Moreover, it provides updates regarding available genetic systems to engineer these microorganisms, in order to use them as platforms for metabolic engineering and production of valuable compounds.

## 2. Heavy Metals

**Figure 1:**
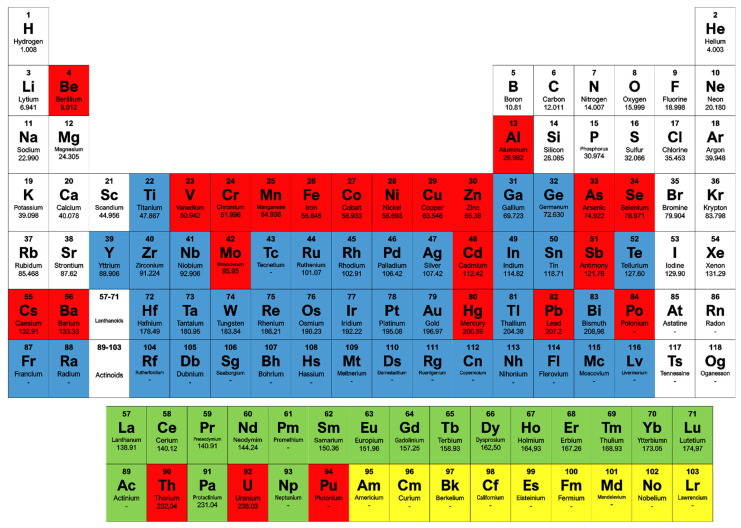
Periodic table of elements. Metals/metalloids are highlighted on the basis of the main characteristic that define them as “heavy”: density > 5 g/cm^3^ (**blue**); toxic (**red**); rare (**green**); synthetic (**yellow**).

The term “heavy metals” is widely referred to a group of metals and metalloids associated with potential toxicity or ecotoxicity. Generally, these metals possess relatively high densities, atomic weights, or atomic numbers. The criteria used for this classification vary, depending on the author and the context [17]; a recent paper reported by the *International Journal of Environmental Research and Public Health*, proposed to refer to them as “potentially toxic elements” [18]. Heavy metals are among the most persistent and toxic pollutants in the environment (Figure 1); they are non-biodegradable, and even in small concentrations, can threaten human and environmental health [19]. Heavy metals naturally occur in soils, rocks, sediments, air, and waters and microbial communities affect their speciation and mobility in the environment, because they are actively involved in metal geochemical cycles [20]. In traces, several heavy metals are essential for life; almost half of all enzymes require the presence of a metal atom to function [21]. Some of them as iron, copper, nickel, manganese, and zinc play key roles as functional centers in proteins and enzymes (i.e., metalloproteins) allowing biological transformations that are exceptionally unlikely to proceed spontaneously [22], as manganese in manganese-peroxidases or copper in laccases [23]; in fact these metallozymes are often employed as industrial biocatalysts (see Section 4.1, Lignin degrading thermozymes). The uncontrolled urbanization and the anthropogenic activities have much altered metal amounts in the environment; in fact, heavy metals are released from mining activities and industrial wastes, vehicle emissions, microplastics floating in the world’s oceans or they come from common devices as lead-acid batteries, fertilizers, paints [24,25,26]. On the other hand, their use is expected to increase over time, since many heavy metals, like copper or nickel, have been identified by the European Commission as critical raw materials for the transition to green energy technologies [27,28].

Microorganisms have evolved resistance systems to cope with these toxic metals that usually rely on a balance between uptake and efflux processes [29]; many of these systems are also common in mesophiles, but in thermophilic Bacteria/Archaea they can present peculiar features [30]. The comprehension of the heavy metals resistance systems in thermophiles is increasingly supported by genome analyses, which allow to individuate their putative molecular determinants [31,32]. To date, at least four mechanisms of heavy metal resistance have been described: extracellular barrier; active transport of metal ions (efflux); enzymatic reduction of metal ions; intracellular sequestration [33,34,35,36,37,38,39]. Some bacteria are able to form complexes or chelates with extracellular polymers that reduce the permeability of metals [40]. However, heavy metals can escape this system and enter the cell thanks to the uptake systems of elements essential for life, for example, arsenic enters the cell via the phosphate or the glucose transporters [41]. Usually, the resistance to heavy metals is due to the coordinated work of intracellular enzymatic oxido-reduction and heavy metal efflux systems which generally consuming ATP, pushes the toxic metal outside the cell [42,43]. The resistance genes are usually organized in operons that also guarantee the expression of a transcription factor that regulates the whole system. Regarding the intracellular sequestration of heavy metals, the general mechanism foresees that some proteins, rich in cysteine residues, form complexes with the metals by exploiting the thiol groups [30].

Sometimes the same microorganism owns more metal resistance mechanisms; for example, *Escherichia coli* possesses either a copper active transport system (CopA) and another system based on multicopper oxidases (CueO) and CusCFBA transport system for periplasmic copper detoxification [23,44]. These resistance mechanisms are often activated as stress response [45,46]; understanding their underpinning molecular basis is crucial for application in the environmental monitoring of metal contamination (biosensing) and/or to set up bioremediation processes [14,47,48].

**Figure 2:**
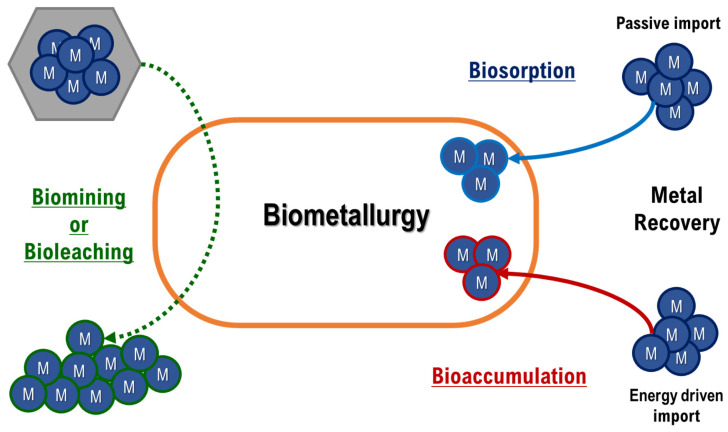
Schematic representation of metal bioprocesses in biometallurgy. Metals (blue circles) can be extracted from ores through biomining/bioleaching (green arrow) and they can be recovered into the cell by passive import (biosorption—blue arrow) or energy driven transport (bioaccumulation—red arrow).

Biometallurgy is the branch of biotechnology that exploits the interaction between microorganisms (or their components) and metals or metal-bearing minerals (Figure 2) [49]. It includes microbial processes as metal biosorption, bioaccumulation or biomining (described below); these processes play a crucial role, on one hand, in the supply of critical raw materials, because can offer eco-efficient alternatives to classical pyro- or hydrometallurgical processes [50,51], and on the other hand in the set-up of strategies for metal biomonitoring and bioremediation (Table 1). In this context, the exploitation of thermophiles offers several advantages related to their ability to survive under harsh conditions and to degrade recalcitrant mineral species. Furthermore, in principle they could be successfully used in situ for metal bioremediation and/or biorecovery in any environment [52].

The possibility to combine metal biorecovery with bioremediation represents an intriguing challenge to reduce process costs: microorganisms can recover metals from polluted sites, contemporarily reducing pollution, and producing valuable elements [62]. The microbial pathways that can be exploited to remove toxic metals from an environment are those related to biosorption/bioaccumulation; they consist into the ability of microorganisms to sequester heavy metals on the cell surface or intracellularly. In particular, the term “biosorption” is referred to passive processes that follow a kinetic equilibrium, while “bioaccumulation” to energy driven processes which require active metabolism [63]. For example, heavy metals metabolic pathways have been widely investigated within the *Geobacillus* genus, since many members of this species are highly tolerant to various heavy metals (As, Ag, Cd, Co, Cr, Cu, Fe, Pb, U, Zn) [57,59,64]. In particular, their biosorption and bioaccumulation mechanisms have been analyzed and applied on environmental samples to remove unwanted metals [3,58,59].

On the other hand, biomining (called “bioleaching” if metals are solubilized during the process) consists in the ability of microorganisms to extract and recover metals from ores and waste concentrates [65,66,67]. Several microbial species, as *Sulfobacillus* sp. and *Ferroplasma* sp., are well known for their ability to solubilize Fe(II) [31,68]; usually they are acidophilic chemolithotrophs (autotrophs or mixotrophs) presenting iron and/or sulfur oxidizing pathways [69]. In some cases, they are used as part of microbial *consortia*, which can perform the bioleaching of different metals simultaneously [53].

Microorganisms able to extract and accumulate metals from ores or geothermal sources are very interesting for their potential application in the biorecovery of rare-earth metals, for example, the Europium (Eu), which is widely used for the production of modern devices (solar cells, mobile phones and computers, biomedical instruments); *Thermus scotoductus* SA-01 can survive in the presence of high levels (up to 1 mM) of Eu, a concentration hundred times higher than that typically found in the environment and is able to extract and accumulate it from geothermal fluids [54]. The biorecovery can be useful also for monitoring metals at low concentration in the environment: Özdemir S. and co-workers [60] set up a preconcentration method with *Bacillus cereus* SO-14 to increase sensitivity in the detection of U(VI) and Th(IV) by ICP-OES (Inductively Coupled Plasma—Optical Emission Spectrometry).

Microorganisms have also been exploited for biomonitoring as *whole-cell* biosensors. In *Thermus thermophilus* HB27, the arsenic responsive transcriptional repressor *Tt*SmtB regulates the expression of the arsenic efflux protein *Tt*ArsX, in particular, *Tt*SmtB responds to variation of Cd(II), As(III) and As(V) concentrations [68,69]; therefore, Antonucci and co-workers engineered *T. thermophilus* to express a reporter gene from the *Ttars*X promoter [55].

In the set-up of systems for metal biomonitoring, it is also possible to follow a decrease of enzymatic activity as a toxicological indicator of heavy metals: Poli and co-workers observed a decrease in the α-amylase activity of *Anoxybacillus amylolyticus*, in the presence of heavy metals [56]. In contrast, Shih-Hung and co-workers followed the inhibition of the iron-oxidizing activity of an *Acidibacillus ferrooxidans* strain [61].

In the fields of heavy metals bioremediation and biomonitoring many thermophilic oxidoreductases have also been characterized. For example, quinone oxidoreductase, chromate reductase, and superoxide dismutase from different *Anoxibacillus* species have been employed for Pb and Cr bioremoval [70,71]; moreover, the arsenate reductase from *T. thermophilus* HB27 (*Tt*ArsC) has been exploited as the biological recognition element for the development of different arsenic biosensors [72,73,74].

## 3. Organic Pollutants

**Figure 3:**
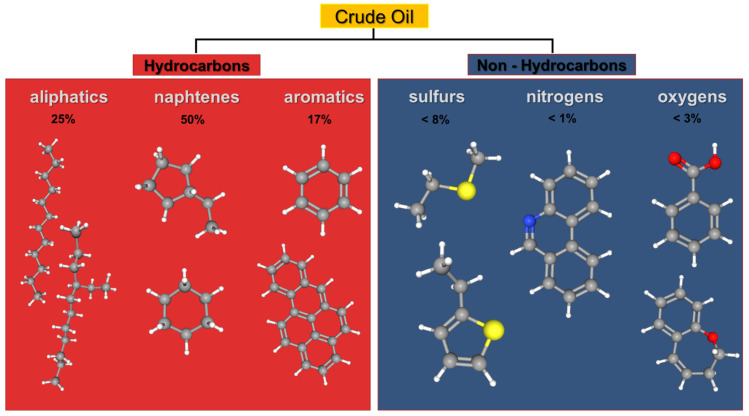
Schematic representation of organic compounds in crude oil. Atoms are reported in grey (C), white (H), yellow (S), blue (N) and red (O).

Organic pollutants are a wide class of chemically different organic compounds released in the environment as toxic wastes [75]. They originate from domestic sewage, urban run-off, industrial effluents, and agricultural wastewater and include pesticides, fertilizers, hydrocarbons, phenols, plasticizers, biphenyls, detergents, oils, greases, and pharmaceuticals [76]. Therefore, the organic pollutants are a very heterogeneous group: the main constituents of the persistent organic pollutants (POPs) are organochlorinated pesticides (OCPs), polycyclic aromatic hydrocarbons (PAHs), polychlorinated biphenyls (PCB), dioxins, and dibenzofurans; they are persistent because they remain intact in the environment for extended periods (years or decades in soil/sediment) [77,78]. These compounds are released in air and soil and even though they are scarcely soluble in water, can be biomagnified in living organisms and cause adverse effects to human health [79]. Among the most common environmental pollutants of the marine environment, there are petroleum hydrocarbons that contaminate the sea through natural oil spills, like reservoirs and volcanic processes in the deep ocean, and artificial oil spills, as oil tanker accidents, oil transportation processes, or oil refineries. In most cases, this last process represents the primary way to contaminate the sea with crude oil (Figure 3) [80,81].

Microbial activities on anthropogenic organic compounds usually arise from evolution of previously existing enzymes and metabolic pathways. Microorganisms have evolved effective catalysts for detoxification of toxic compounds, as result of a selective pressure [82]. Generally, toxic compounds are converted into metabolites entering central metabolic pathways: for example, pathways responsible for the biodegradation of aliphatic and alicyclic carboxylic acids include β-oxidation, combined α- and β-oxidation, and aromatization pathways [83]. In *Sulfolobus solfataricus* members of the multiple antibiotic resistance regulators family (MarR-family) are involved in detoxification of aromatic compounds, as benzaldehyde and salicylate [84,85]. Aromatic hydrocarbon dioxygenases, belonging to the large family of Rieske non-heme iron oxygenases (ROHs), catalyze the initial reaction in the bacterial biodegradation of a diverse array of aromatic and polyaromatic hydrocarbons, aromatic acids, chlorinated aromatics, and heterocyclic aromatic compounds [86]. They are attractive in biotechnology for bioremediation as well as for the production of industrially and medically important chiral chemicals; for example, toluene dioxygenase catalyze the oxidation of benzene to benzene *cis*-diol [87], and many thermophilic bacteria distributed mainly among *Chloroflexi*, *Deinococcus–Thermus,* and *Firmicutes* have been identified as sources of these appealing enzymes [88].

Bioremediation of toxic compounds represents an effective and sustainable technology, compared with physical and chemical remediation technologies, based on microbial activities that in an ideal bioprocess degrade all the substances to CO_2_ and H_2_O (complete mineralization) [89]. In crude-oil bioremediation processes, the exploitation of thermophiles can be considered an optimal choice; in fact, at higher temperature there is a decrease in the oil viscosity that increases the diffusion rates of organic compounds making them more accessible to microbial degradation [90]. Since the degradation activity can be often substrate-specific, biodegradation processes can be optimized using microbial consortia, thus expanding the spectrum of action [91,92]; for example, two strains of *Geobacillus jurassicus* and *Geobacillus subterraneus*, isolated from the Dagang high temperature oil field in China, can grow on benzoate but not phenol; therefore, to degrade crude oil, they need the presence of complementary phenol degrading activities (in this specific case a *G. stearothermophilus*, which can use phenol but not benzoate) [93].

In addition to biodegradation processes (Table 2), thermophiles also produce macromolecules that can be considered useful for the bioremediation of organic pollutants: *Bacillus licheniformis* and *Anaerophaga thermohalophila* have been characterized for the production, under anaerobic conditions, of low molecular weight peptides, which are surface-active compounds, exploitable as biosurfactants for oil removal [94].

The substrate specificity and the stability of detoxifying thermozymes make them also exploitable as recognition elements of biosensors, especially those which require electrochemical detection; for example, the haloacid dehalogenase, L-HAD_ST_ of *Sulfolobus tokodaii* was immobilized an N-hydroxysuccinimidyl Sepharose resin and used for the detection of halogenated organic compounds, retaining 70% of its initial activity after storage at 4 °C for 6 months [95,96].

### Toxic Dyes

Industrialization has represented one of the main causes of water pollution. Wastewaters can be rich in recalcitrant, mutagen and carcinogenic compounds [97]. Dyes are a class of very toxic pollutants that are released in the wastewaters of textile manufacturing. These recalcitrant compounds change both the pH and the chemical oxygen demand (COD) and biochemical oxygen demand (BOD) in aquatic ecosystems [98,99]. The dyes are classified on the basis of their chemical structure or industrial uses. The most employed dyes are acid dye, synthetic dye, and direct dye. Even if they can are complex organic molecules, each dye has a characteristic chromophore: for example, the acid and synthetic azo dyes are typical for their azo linkage (–N = N–), the central chromophore of the anthraquinone dyes derives from the oxidation of anthracene, the indigoid dyes derive from indoles [100].

There are several technologies for colored wastewater remediation: physical, chemical, and biological. The physical treatments include screening, coagulation, precipitation, adsorption and membrane filtration; the chemical treatments comprise coagulation–flocculation, oxidation, ozonation, Fenton oxidation, photocatalytic oxidation, ion exchange, and electrochemical treatments; the biological methods are aerobic, anaerobic, and anaerobic–aerobic treatments in which the contaminated organic compounds are converted into safe and stable compounds [101,102]. Each technology has advantages and inconveniences; in fact, to date the typical method for wastewater remediation of colored waters is physical–chemical flocculation combined with biological treatment [101,102].

Interestingly, the analysis of microbial communities of these waters revealed the occurrence of several bacteria able to decompose the dyes: they possess intra/extra cellular oxidoreductases, such as dye decoloration peroxidases (DyP), laccases, azoreductases [103]. Many laccases and azoreductases have been characterized in several thermophiles like *T. thermophilus*, *Geobacillus, Anoxibacillus, Thermosediminibacter* species, and others (Table 3). Their enzymes are expected to be more stable to extreme temperatures and pHs in comparison to those of mesophilic bacteria.

In the genome of *T. thermophilus* HB27, a laccase that is able to oxidize six different dyes (dye orange, acid red dye, green dye, naphthol brilliant blue, Remazol brilliant blue, Congo red), has been characterized; the enzyme requires an electron shuffle, that supports this reaction for some dyes [107]. Also *Geobacillus* sp. JS12 contains a laccase, LacG, that can decolor these artificial compounds at 70 °C [110]. In the alkaliphilic and thermophilic bacterium *Anoxybacillus* sp. strain UARK-01, the UARK 01 laccase can oxidize the Congo red substrate, one of the most toxic dyes [105]. In addition to the laccases, these extremophiles also have a striking azoreductase, active on different dyes; for example, *Anoxibacillus* sp. PDR2 acts towards the direct black G [113]; moreover, the degradation of the same dye can be obtained by a thermophilic microflora, consisting of facultative aerobic (*Anoxybacillus flavithermus* strain 52-1A*, Tepidiphilus thermophilus* strain JHK30, *Tepidiphilus succinatimandens* strain 4BON, *Brevibacillus aydinogluensis* strain PDF25, *Bacillus thermoamylovorans* strain DKP and *Geobacillus thermoleovorans* strain NP1) and exclusively anaerobic bacteria (*Thermoanaerobacterium thermosaccharolyticum* strain DSM 571, *Thermoanaerobacterium thermostercoris* strain Buff, and *Caloramator proteoclasticus* strain Uruguayensis) [114]. These examples give a generic view on the potential application of thermophilic oxidoreductases in biological detoxification.

## 4. Lignocellulosic Biomasses

In the last decades, there is a growing interest in the use of microbes in industrial processing to break waste food and lignocellulose biomasses to produce biofuels and bioproducts. Among renewable resources, non-food lignocellulosic waste biomasses are currently considered among the most promising materials, since they are present in large quantities and at low cost [115]. Every year, a significant amount of lignocellulosic residues is generated worldwide from agricultural wastes, food industry, household garbage, non-food seeds, etc. (see Table 4), causing an increase in environmental pollution. Lignocellulosic wastes are also often improperly stored and recalcitrant to different disposal treatments; moreover, when burnt, they provoke environmental pollution problems. Thus, the re-use and exploitation of such wastes in industrial biotechnology to produce interesting chemicals allows to bypass a part of disposal treatments [116].

Lignocellulose is a significant component of plant biomass and it consists of cellulose, hemicellulose, and lignin. Cellulose and hemicellulose are polymers of different sugars; in particular, the principal constituent of lignocellulosic biomass is cellulose, a polysaccharide composed of β-1,4-linked D-glucose units, widely employed for paper and cardboard production. Instead, hemicellulose is a complex branched polysaccharide formed by a mixture of xylans, mannans, β-glucans, and xyloglucans, depending on the type of wood. In softwood, hemicellulose mainly consists of galactoglucomannan, composed of β-1,4-linked D-glucose and D-galactose units. In contrast, xylan is the constituent of hemicellulose in hardwood, and it is formed of β-1,4-linked D-xylose units, which can be substituted with other monosaccharides [119]. Hemicellulose is closely associated with cellulose filaments and covalently attached to lignin, forming a matrix.

Lignin is an aromatic heteropolymer composed of ether and C–C bonds that link phenylpropanoid aryl-C3 units. The percentage composition of these polymers in waste lignocellulosic biomasses varies as shown in the table below; the content of cellulose, hemicellulose and lignin also changes in a single plant depending on the age, stage of growth, and other conditions [120].

**Figure 4:**
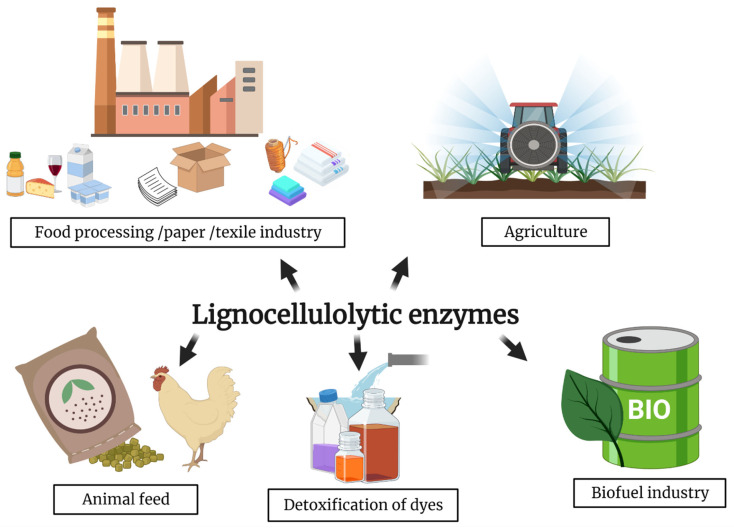
Industrial applications of lignocellulolytic enzymes. In food processes cellulases and xylanases are employed to improve the shelf life of dairy products and to hydrolyze monosaccharides in milk processing; they are also used in winery industries and to decrease viscosity of fruit juice. Also, laccases are used to increase quality of beverages and food, for example eliminating toxic substances. In pulp/paper industry laccases and xylanases enhance pulp bleaching for paper manufacturing, and cellulases improve flexibility and softness of fibers. Lignocellulolytic enzymes can be employed for improving nutrient digestibility of animal feeds, for enhancing color and surface brightness of fabric in textile industry, for textile dye bleaching, for synthesis of complex polymers. In agriculture, they are involved in fruit ripening and defense mechanisms against insects. Laccases are employed in wastewater treatment of colored waters. Lignocellulolytic enzymes are used in biorefinery systems to produce biofuels [103,127,128] (Created with BioRender.com (accessed on 20 January 2021)).

A high number of microorganisms, belonging to both bacterial and archaeal kingdom, possesses complex metabolic pathways able to decompose lignocellulose. Their enzymes can be utilized as biocatalysts for green approaches in several industrial fields, such as paper and pulp industry, food processing and textile sector, agriculture, animal food production, etc. (Figure 4). Furthermore, several microbial based technologies for the exploitation of lignocellulosic wastes as raw materials for producing bioproducts and biofuels were set up. In the specific case of bioethanol production, from early 70 to 2000s, a “first-generation technology” (1G) was developed, in which the biorefinery systems were based on the use of starch/sugar crops (sugar beet, maize, and sugar). However, 1G has several unsustainability issues bound to the great request of crops subtracted to the food chain and the cultivation of large areas (destined for this purpose) that causes deforestation and decrease of biodiversity [121]. As an alternative, in the “second-generation” technologies (2G), lignocellulosic materials are employed as feedstocks; in this respect, lignocellulose is cheap and immediately available in a large amount [122], but the development of tailored technologies is necessary to exploit more recalcitrant components. For example, both 1G and 2G technologies have to simultaneously maximize production yield and reduce costs and environmental impact; in both cases, exploitation of microbial mechanisms and biocatalysis supports the process. The bioethanol production process consists of different phases: biomass pre-treatment, saccharification, fermentation, and distillation. The main difference between the two technologies lies in the complexity of the starting raw material; in the first-generation technologies after pre-treatment of sugarcane and maize, a chemically homogenous material (sucrose and starch) can be easily broken into sugar units by a limited number of enzymes like amylases, amylopullulanases or glucosidases [123]. For the improvement of 1G processes, investigation on α-amylases, α-D-glucosidases, pullulanases and amylopullulanases of thermophilic bacteria and archaea have been carried out. For example, *T. thermophilus* HB27, *Thermoanaerobacter ethanolicus* 39E, *Geobacillus thermoleovorans* NP33, *Rhodothermus marinus, Clostridium thermosulfurogenes, Clostridium thermocellum, Desulfurococcus mucosus, Fervidobacterium pennavorans*, *Bacillus stearothermophilus*, *Thermotoga maritima* and some species of the genera *Pyrococcus, Thermoanaerobacter,* and *Thermococcus* have been studied since they are able to produce starch degrading enzymes. However, some of their biocatalysts have limited activity with high starch concentration (>30%). Therefore, mesophilic hosts are still the preferred ones for bioethanol production [124,125]. In fact, standardized methodologies for saccharification and fermentation have been mainly optimized in engineered mesophilic yeasts or microbes (*Saccharomyces* and *Zymomonas* spp.) with a high bioethanol production yield [126].

On the other hand, the degradation of lignocellulose is more complicated because the starting matrix is heterogeneous. The lignin removal step requires a significant amount of energy (acid hydrolysis or steam explosion) to release sugar polymers for the subsequent saccharification step [123]. Moreover, in 2G, either in saccharification and fermentation, additional steps are necessary to achieve the complete production of bioethanol from cellulose and hemicellulose; therefore, despite its cheapness and availability, lignocellulosic material implicates a more elaborate treatment process.

In this context, the use of thermophiles in lignocellulosic biomass degradation has the advantage that higher temperatures and organic solvents can be used, reducing either risks of microbial contamination or energy consumption (because the cooling steps are not necessary), and increasing rates of hydrolysis and product yields [129]. Another attractive progress on the use of renewable lignocellulosic biomass to produce bioethanol or feedstock chemicals consists in setting up microbial based bioprocesses that exploit the synergistic degradative capabilities of thermophilic microorganisms or consortia [130].

### 4.1. Lignin Degrading Thermozymes

Enzymes that depolymerize lignin are isolated principally from the white-rote fungi; some of these enzymes are manganese peroxidase (MnP), lignin peroxidase (LiP), versatile peroxidase (VP), and laccase. In particular, laccases are a heterogeneous subfamily of multicopper oxidases (MCOs) that can be involved in several biological processes like lignolysis and detoxification of dyes (see above) [131].

Recent studies have described very promising thermostable laccases able to degrade lignin derived from thermophilic microorganisms such as *Bacillus* sp. PC-3 and sp. FNT with optimum activity temperatures of 60 and 70 °C, respectively [132,133]. The first archaeal laccase was characterized from the halotolerant *Haloferax volcanii,* a promising microorganism for the lignin break-down purposes*;* this archaeon grows up to 50 °C and it possesses a stable glycoprotein, the laccase LccA that acts on several substrates at elevated temperature (55 °C), high salt concentrations (0.1 to 1.4 M) and it can maintain its activity also in organic solvents [134]. Also, many *Thermus* specie*s* have laccases that can be employed in lignin degradation; laccase of *Thermus* sp. 2.9 can retain 80% of its activity at 70 °C for 16 h and is able to successfully delignify *Eucalyptus* biomass [135]. Two strains of *T. thermophilus* (HJ6 and HB27) produce laccases capable of maintaining an optimal temperature range of activity at 85–90 °C for reactions up to 1 h [107,136]. Moreover, two hyperthermophilic bacteria present laccases with remarkable heat stability; a chemolithoautotrophic bacterium, *Aquifex aeolicus,* expresses a multicopper oxidase with an optimal temperature of 75 °C but that preserves its activity at 80 and 90 °C for up to 9 and 5 h, respectively [137]. Furthermore, the laccase-like multi-copper oxidase of *Thermobaculum terrenum* is extremely thermostable with a half-time of inactivation of 2.24 days at 70 °C and 350 min at 80 °C and pH 7 [138].

These features suggest that an impressive compromise between thermostability and lasting activity exists for these enzymes; for this reason, they are considered as promising tools to degrade the lignin component of biomasses.

### 4.2. Cellulose and Hemicellulose Degrading Thermozymes

Several thermophilic archaea and bacteria are able to produce a considerable amount of promising cellulose/hemicellulose degrading enzymes. These thermophilic enzymes, differently from their mesophilic counterparts, have the advantage that they can be added immediately after the thermochemical pre-treatment of biomass, making the cooling steps not necessary, increasing conversion efficiency, and saving time [139].

Whereas cellulose can be completely depolymerized through endoglucanases, exoglucanases and β-D-glucosidases, the total degradation of hemicellulose requires a wider pool of enzymatic activities (i.e., β-xylosidases, β-xylanases, β-glucuronidases, *β*-mannanase, *β*-mannosidase, *α*-galactosidase, etc.).

Several thermophilic cellulose degrading bacteria have been isolated from distinct environments like hot springs, compost systems and soil. They include different species belonging to the genera *Actinomadura, Alicyclobacillus*, *Anoxybacillus, Acidothermus, Bacillus, Caldanaerovirga, Caldicellulosirupto, Cellulomonas, Clostridium, Dictyoglomus, Geobacillus, Paenibacillus, Nesterenkonia, Paenibacillus, Pyrococcus, Rhodothermus, Sulfolobus, Thermoanaerobacterium,* and *Thermotoga*; they can produce both cellulose and hemicellulose degrading enzymes that can raise the rates of biomass hydrolysis if they are used in industrial bioprocesses [140].

Some examples of remarkable thermophiles include the following ones.

*Acidothermus cellulolyticus* 11B, isolated from a hot spring in Yellowstone National Park, produces a tri-functional enzyme that can break down birchwood xylan with high efficiency; in fact, this enzyme has endo-xylanase, arabinofuranosidase, and acetyl-xylan esterase activities [141].

Two thermoalkaline species of *Anoxybacillus (kamchatkensis* NASTPD13 and sp. 3M) express many xylanases and β-xylosidases, respectively, highly resistant to alkaline and acidic pHs, denaturing agents and organic solvents [142,143]. Also the facultative anaerobic *Bacillus coagulans* MA-13, which lives at an optimal temperature of 55 °C, secretes an endo-1,4-β-glucanase which can act from 37 to 60 °C. *B. coagulans* MA-13 is also able to ferment sugars derived from pre-treatment of lignocellulose to lactic acid in the presence of inhibitors; in fact, it was proved that this bacterium can grow and ferment in bioreactors containing 95% hydrolysate [144]. At the same time, seed culture pre-adaptation of *B. coagulans* MA-13, before simultaneous saccharification and fermentation step, can improve the production of lactic acid; again, it has a pool of interesting intra- and extracellular enzymes with glycosyl hydrolyzing activities that make *B. coagulans* MA-13 useful for increasing nutritional value of food [145,146].

*Clostridium thermocellum* has a non-enzymatic scaffolding protein bound with different enzymatic subunits that simultaneously degrade cellulose and hemicellulose [147]. In 2018 a new cellulolytic strain was identified in the *Chryseobacterium* genus, which produces an enzyme with a double cellulase/xylanase activity working either on carboxymethylcellulose and birchwood xylan [148].

Instead, the anaerobic *Caldicoprobacter* sp. CL-2, isolated from bovine manure compost, has a xylanase activity showing a modular structure with a glycoside hydrolase domain coupled with a carbohydrate binding module [149]. Another hyperthermophilic microorganism from geothermal springs that produce miscellaneous glycoside hydrolases is *T. maritima*; it has an endoglucanase enzyme (Tm_Cel5A) with an optimum T of 80 °C and pH of 4.8. Tm_Cel5A is a peculiar GH5 (glycoside hydrolase family 5) enzyme with an unusual activity because it can act both on glucan and mannan based polysaccharides, while the other GH5 hydrolysing enzymes degrade either cellulose or mannans [150].

*Dictyoglomus turgidum* is another thermophilic microorganism that displays a set of genes encoding putative enzymes with glycosyl hydrolyse activity; this not yet well characterized thermophile has an endo-1,4-β-mannanase, *Dtur*CelB, with a high thermoresistance (Tm of 88 °C) and a good thermal and pH stability; it is also resistant to chemicals and has been analyzed in an enzymatic cocktail able to cut-off cellulose and hemicellulose [151,152]. In fact, different thermophilic biocatalysts can be utilized synergistically for the complete breakdown of hemicellulose sugars (pentose and hexose) of lignocellulosic material. The two recombinant thermophilic enzymes, the above mentioned *Dtur*CelB from *D. turgidum* and the α-galactosidase from *T. thermophilus*, can be tested in the lignocellulose pre-hydrolyzing step right before the saccharification step [153].

Other two thermophiles, *Thermotoga neapolitana* 5068 and *T. thermophilus,* display the most thermoactive (~100 °C) and thermostable (half-life of 30 h at 70 °C) *α*-galactosidase activities, respectively [154,155].

Archaeal glycoside hydrolyzing enzymes have also been exploited to improve biomass degradation processes. For example, the hyperthermophilic archaeon *Sulfolobus shibatae* encodes an endo-1,4-β-D-glucanase that accomplishes the break-down of carboxymethylcellulose, xylan and barley β-glucan [156]. *Pyrococcus furiosus* produces extracellular endoglucanases, intracellular glucosidases, and different intra- and extracellular amylases with a high thermostability in the range 80–100 °C; one of these enzymes, a β-glycosidase, is immobilized and used in industrial process of lactulose production [157]. Furthermore, *Saccharolobus solfataricus* expresses a membrane-bound xylanase, an extracellular endoglucanase, intra- and extracellular galactosidases, an extracellular xylosidase and an intracellular mannosidase. In the case of *S. solfataricus*, its thermozymes have been used as model for engineering mesophilic enzymes in order to improve their thermostability; for instance, the β-glycosidase of this archaeon, that shows a maximal activity above 95 °C, represents a fine example of an efficacious heterologous production in a yeast expression system [158].

In recent years, several studies are focused on taking advantage on thermophilic communities that can provide a high hydrolyzation rate of lignocellulosic material (Table 5). For example, consortia formed by bacterial and fungal microorganisms such as Alcaligenaceae*,* Burkholderiacea*, Thermoamylovorans,* Xanthomonadaceae*, Mycobacterium, Talaromyces* and *Rubrobacter* can decompose biomasses with a high content of lignin [159].

Furthermore, high throughput genome sequencing, transcriptomics, proteomics, metagenomic, and other omics techniques together with metabolic engineering strategies and bioinformatic tools have contributed significantly to explore a considerable amount of novel thermophilic lignocellulolytic microorganisms and enzymes.

The development of genome editing tools also represents a new approach to address biomass degradation by microorganisms; in fact in a next future, the genome manipulation of thermophilic bacteria will make possible to develop fine bioprocessing microbial strains, that will be capable of better performing degradation of lignocellulose [163]. Thus, thermophiles have a great potential to be considered as a suitable platform for metabolic engineering to produce various biomolecules and/or valuable chemicals from lignocellulosic biomasses.

## 5. Engineering of Thermophiles

Thermophilic microorganisms have unique biochemical and physiological characteristics with important biotechnological implications. Thermophilic microorganisms can be used in numerous applications, such as biocatalysis, or as sources of thermoactive or thermostable enzymes. However, unfortunately, their employment as *whole-cell* systems is limited by the lack of easily usable genetic systems. This situation has changed recently, with unprecedented progress in genetic tools for extremophilic microorganisms, and the use of these microorganisms as platforms has become possible.

Significant studies have been made to develop and improve molecular genetic techniques for thermophilic microorganisms in the past decade, either belonging to the bacterial or archaeal kingdom. A significant challenge for genetic modification in thermophiles is the choice of a selectable marker to screen positive transformants. The antibiotics typically used in mesophiles often target cell components specific to bacteria and are ineffective against the archaeal species. Even in cases where antibiotics are useful, both the antimicrobial compound and the gene product that confers resistance must be stable at elevated temperatures. Due to the low efficiency of the heat-resistant antibiotic selection markers, usually nutritional selection systems such as enzymes essential for the synthesis of amino acids can be used. To date, genetic techniques have been obtained for ten such archaea, including *Metallosphaera*, *Sulfolobus*, *Thermococcus,* and *Pyrococcus* species [164,165,166,167].

The creation of genome editing tools enabling stable integration of genetic elements into host chromosomes is crucial for industrial applications, where plasmid instability becomes problematic and volumes of antibiotics on an industrial scale are very polluting. In principle, two alternative approaches for developing genome editing tools can be adopted for thermophilic bacteria; one is by adapting mesophilic protocols to function at elevated temperatures. The second is to seek alternative means of genome editing from thermophilic springs. Several examples describe the use of homologous recombination to knock out or replace chromosomal genes in thermophilic bacteria. In 2012, Suzuki and co-workers developed a *pyr*F/*pyr*R counterselection system for *Geobacillus kaustophilus*, enabling marker-free genome editing at 60 °C [168].

Another widely used system to obtain genetic manipulation of thermophilic bacteria is the *Cre/loxP* site-specific recombination [169]. This recombination is performed between two *loxP* sites using a Cre recombinase, *loxP* is a 34 bp consensus DNA sequence with a central spacing region of 8 bp, which defines its orientation, flanked by two 13 bp palindromic sequences, which are the Cre binding sites. The *Cre/lox* system’s effectiveness in a broad spectrum of biological species and a wide variety of applications has made this technology indispensable for in vivo genetic manipulation. This system allows various recombination types, such as conditional recombination, intermolecular recombination and time and space specific recombination [169]. Recently, a *Cre/lox* system was developed for the thermophilic bacterium *T. thermophilus* HB27 [170], leading to the development of a highly efficient method of destroying multiple genes to facilitate genetic manipulation of this bacterium. The most important advantage that made easier to develop genetic tools for *T. thermophilus* is the constitutive expression of a natural competence system in several strains [171]. Several plasmids have been developed to transform *T. thermophilus*, and some of these, suitably modified using regions of homology to the chromosome, have been used to stimulate homologous recombination, obtain deletions of genes, thus allowing the study of the in vivo function of specific proteins [172].

For these reasons, *T. thermophilus* is considered a biological model for functional studies and a right candidate for biotechnological applications. However, its efficient defense system against the exogenous DNA can be an impairment since it can destroy the cloning vectors used for transformation*;* in 2014, Daan C. Swarts and co-workers identified *Tt*Ago, a protein belonging to the Argonaute family as the protein responsible for the prevention of the uptake and propagation of foreign DNA [173]. The researchers observed that the protein generally attacks the AT-rich regions of double-stranded DNA, leading to the complete plasmid degradation by other nucleases [173].

### The Rise of the CRISPR-Cas Era

**Figure 5:**
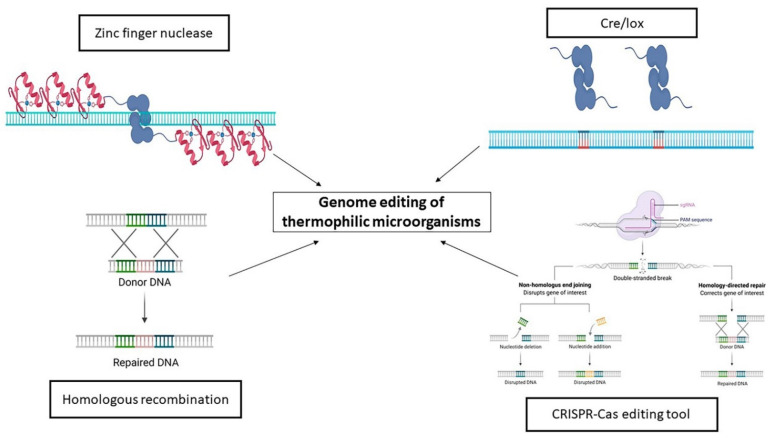
Schematic representation of the genome editing tools for thermophilic microorganisms. The Zinc finger nuclease is the first genome editing tool setup for thermophilic microorganisms. The most common is the spontaneous homologous recombination. In the last decades, the Cre/lox and CRISPR-Cas based tools were adapted for thermophiles (Created with BioRender.com (accessed on 20 January 2021)).

Until 2013, the principal genome editing tools were the zinc finger nucleases, the transcription activator-like effector nucleases and intrinsic homologous recombination systems [174,175,176]. These systems use artificial fusion proteins composed by an engineered DNA-binding domain fused to the non-specific nuclease domain of the restriction enzyme FokI. These systems were extensively used for the genome editing of eukaryotic microorganisms. A new technology for genome editing rose based on RNA-guided engineered nucleases (CRISPR-Cas9 system) in the last decades. Although the CRISPR array was discovered in the late 1980s [177], its function remained unknown until 2005 [178]. Only in 2007, it was concluded that it represented a bacterial innate immunity system [179,180]. The transition of the CRISPR/Cas system from a biological phenomenon to a tool for genome engineering occurred when it was shown that the target DNA sequence could be reprogrammed simply by changing 20 nucleotides in the crisprRNA (crRNA) and that the targeting specificity of the crRNA could be combined with the structural properties of the tracrRNA (trans-activating crisprRNA) in a chimeric single guide RNA (sgRNA) [181] (Figure 5).

Furthermore, the evidence that sgRNAs with different specificities could be produced made it possible to modify more loci simultaneously, giving a connection to the so-called CRISPR-mania [182]. The various genome editing applications pioneered in human and animal cells have recently been transferred back to bacteria to carry out genome editing and transcriptional control, as well as genome-wide screens. In fact, the CRISPR-Cas system was used to obtain some genetically modified prokaryotes. However, one problem for applying this genetic editing tool to thermophilic microorganisms, is that it is based on a mesophilic system. In recent years the research has been going towards the search for Cas proteins from thermophiles; in fact, a thermostable genome editing tool was developed based on a thermophilic Cas9, that can be used up to 55 °C and contains everything necessary for genome editing in a single plasmid; with the advent of the ThermoCas9, genome manipulation in moderate thermophilic bacteria becomes possible, making the editing process much more comfortable and less time-consuming [183].

A genome-editing tool was recently developed for moderate thermophilic bacteria obtained using the Cas12a from *Francisella novicida* [184]; this system allowed to obtain knockout mutants in less than one week with high editing efficiencies. *Fn*Cas12a has an interesting potential for the genome editing of many thermophilic bacteria and archaea.

Cas9 and Cas12a are multidomain CRISPR-associated nucleases that can cleave complementary DNA targets using a guide RNA. The Cas9 belongs to type II-a, while the Cas12a to type V-a. The first enzyme is the best characterized and utilizes nuclease for genome-editing purposes. In the last years, Cas12a has emerged as a potential alternative. These two enzymes have distinct evolutionary origins and present different structural architectures, resulting in specific molecular mechanisms; in fact, the nuclease activities of Cas9 and Cas12a and the resulting DNA repair outcomes are affected by circumstantial factors such as cell type, target sequence, and genomic context [185]. Their biological differences influence their application as genome editing tools: in some cases, the Cas9 activity is more suitable for some organisms, in other cases the best option is to use Cas12a. Instead, Cas9 and Cas12a and their engineered variants are highly complementary in their properties and together build up a powerful and versatile toolkit.

## 6. Conclusions

Extremophiles represent a class of microorganisms very interesting for their ability to live in harsh conditions, not only high temperature, but also extreme pHs and high salinity concentrations. These peculiar characteristics make them and their biocatalysts very promising tools for industrial and environmental applications. Thermophilic extremophiles stand out in biometallurgy for biomonitoring and bioremediation, as well as in degradation of organic biomasses to transform them into resources ready to be re-used. In fact, in addition to the biofuel production, hydrolyzing extremozymes have a wide range of applications in the food, feed, beverage, textile, pulp and paper industry.

Improved knowledge in *omic-era* and the increasing need to address environmental pollution with green processes drive biotechnological research in search of microorganisms that can replace chemical processes. The newly available thermophilic genome editing tools based on the CRISPR-Cas system, open the way for the complete achievement of these goals in various industrial fields. In fact, the rise of the “CRISPR-Cas *era*” makes possible the application of engineered extremophiles as a *whole-cell* platform.

## Figures and Tables

**Table 1:** Examples of thermophiles exploited in biometallurgy.

Heavy Metals Tolerant Thermophiles				
Application	Target	Microorganism	Temperature	Ref.
Bioleaching	Cu, Zn, Ni, Cd, Al, Cr, Pb	Consortium of *Sulfobacillus thermosulfidooxidans* and *Thermoplasma acidophilum*	45 °C	[53]
Biosorption	Ag, Cd, Co, Cr, Cu, Fe, Pb, Zn	*Geobacillus thermodenitrificans*	60 °C	[3]
Biomineralization and Bioaccumulation	Eu	*T. scotoductus* SA-01	65 °C	[54]
Biosensing	Cd, As	*T. thermophilus* HB27	70 °C	[55]
Biosensing	Ni, Zn, Co, Hg, Mn, Cr, Cu, Fe, Cd	*A. amylolyticus*	60 °C	[56]
Biosorption	Cd, Cu, Co, Mn	*Geobacillus thermodenitrificans* and *A. amylolyticus*	60 °C	[57]
Biosorption	Cd	*Geobacillus stearothemophilus*	60 °C	[58]
Bioaccumulation	Cd, Cu, Ni, Mn, Zn	*Geobacillus toebii* subsp*. Decanicus* *Geobacillus thermoleovorans* subsp*. stromboliensis*	60 °C	[59]
Biosorption	U, Th	*B. cereus* SO-14	65 °C	[60]
Biosensing	As, Cd, Hg, Pb	*Acidibacillus ferrooxidans*	45 °C	[61]

**Table 2:** Examples of thermophiles exploited in bioremediation processes of organic compounds.

Organic Compounds Degrading Thermophiles				
Bioprocess	Target	Microorganisms	Temperature	Ref.
Biodegradation	Crude oil	Consortium of *Bacillus, Geobacillus* and *Clostridium*	55 °C	[91]
Biodegradation	Hydrocarbons	*Geobacillus pallidus*	30–70 °C	[4]
Biofilter	Volatile Organic Compounds (VOCs)	Consortium of 25 genera belonging to Alphaproteobacteria, Betaproteobacteria, Gammaproteobacteria, Deltaproteobacteria, Flavobacteriia, Sphingobacteriia, and Bacilli classes	50–60 °C	[92]
Biodegradation	Phenolic compounds	*Bacillus thermoleovorans* sp. A2	65 °C	[95]
Biodegradation	Hydrocarbons	Consortium of *Geobacillus* and *Thermoattinomyces* spp.	60 °C	[93]

**Table 3:** Examples of dye-decolorizing thermophiles.

Microorganisms	Substrates	Ref.
*Anoxybacillus pushchinoensis, Anoxybacillus kamchatkensis and Anoxybacillus flavithermus*	Reactive Black 5	[104]
*Anoxybacillus* sp.	Congo red	[105]
*G. stearothermophilus*	Remazol Brilliant Blue R, Methyl Orange, Malachite Green (MG) and Indigo Carmine	[106]
*T. thermophilus* HB27	Dye orange, Acid red dye, green dye, naphthol brilliant blue, Remazol brilliant blue, congo red	[107]
*T. thermophilus* SG0.5JP17-16	Congo Red, Reactive Black B and Reactive Black WNN, and Remazol Brilliant Blue R	[108]
*Thermus* sp. 2.9	Xylidine, RBBR, Gentian Violet, Methyl Orange	[109]
*Geobacillus* sp. JS12	Congo red, Malachite green	[110]
*Anoxybacillus ayderensis* SK3-4	Direct blue 6, acid black 1, direct green 6, direct black 19, and acid blue 93	[111]
*Thermosediminibacter oceani*	Malachite green (MG) and Congo red	[112]

**Table 4:** Percentage content of most common lignocellulosic wastes [117,118].

Lignocellulosic Wastes	Lignin (%)	Hemicellulose (%)	Cellulose (%)
Softwood stems	25–35	25–35	45–50
Hardwood stems	18–25	24–40	40–55
Miscellaneous Corn stover	19	22	39
Wheat straw	15	50	30
Rice straw	18	24	33
Nutshells	30–40	25–30	25–30
Peels	14–20	11	4
Shells	26–30	20–25	40–45
Sorted refuse	20	20	60
Swine waste	n/a	28	6
Solid cattle manure	2.7–5.7	1.4–3.3	1.6–4.7
Grass	10–25	35–50	30–40
Cotton seed hairs	0	5–20	80–95
Leaves	0	80–85	15–20
Sawdust	14–34	71–89	31–64
Paper	0–15	0	85–99
Newspaper	14–19	25–40	40–55
Wastepaper from chemical pulps	5–10	10–20	60–70
Primary wastewater solids	24–29	n/a	8–15

**Table 5:** Examples of thermophilic bacteria and archaea able to hydrolyze the lignocellulose.

Lignocellulosic Component	Microorganism	Temperature	Ref.
**Lignin**	*A. aeolicus*	89 °C	[137]
	*Bacillus* sp. PC-3	55–92 °C	[132]
	*Bacillus* sp. FNT	50–55 °C	[133]
	*H. volcanii*	50 °C	[160]
	*Thermus* sp. 2.9	65 °C	[135]
	*T. thermophilus* HJ6	80 °C	[136]
	*T. thermophilus* HB27	70 °C	[107]
	*T. terrenum*	67 °C	[138]
	*Fungal and bacterial consortium*	55 °C	[159]
**Cellulose and hemicellulose**	*A. cellulolyticus* 11B	70 °C	[141]
	*A. kamchatkensis* NASTPD13	60 °C	[142]
	*Anoxybacillus* sp. 3M	55 °C	[143]
	*B. coagulans* MA-13	55 °C	[144]
	*Brevibacillus borstelensis* SDM	50 °C	[161]
	*C. thermocellum*	60 °C	[147]
	*Chryseobacterium* sp.	55 °C	[148]
	*Caldicoprobacter* sp. CL-2	60–75 °C	[149]
	*D* *. turgidum*	75–80 °C	[151]
	*T. maritima*	80 °C	[150]
	*T. neapolitana* 5068	70–80 °C	[162]
	*T. thermophilus* HB27	70 °C	[107]
	*P. furiosus*	100 °C	[158]
	*S. shibatae*	80 °C	[156]
	*S. solfataricus*	80 °C	[158]

## Data Availability

Not applicable.
